# Nitrogen, phosphorus, and potassium requirements to improve *Sideritis cypria* growth, nutrient and water use efficiency in hydroponic cultivation

**DOI:** 10.1016/j.heliyon.2024.e40755

**Published:** 2024-12-04

**Authors:** Antonios Chrysargyris, Nikolaos Tzortzakis

**Affiliations:** Department of Agricultural Sciences, Biotechnology and Food Science, Cyprus University of Technology, 3603, Limassol, Cyprus

**Keywords:** Antioxidants, Nutrient uptake, Soilless culture, Tailoring nutrient solution, Macronutrients

## Abstract

Medicinal and aromatic plant (MAP) production is gaining popularity for industrial agriculture, with phytochemical compounds having a significant impact on human health. Plant fertilization must be carefully considered as it is strongly affecting the biochemical profile of MAPs. The present study examined the *Sideritis cypria* responses to different nitrogen (N: 75, 150, and 300 mg/L), potassium (K: 150, 350, and 550 mg/L), and phosphorus (P: 50, 75, and 100 mg/L) concentration in the nutrient solution (NS) in hydroponics. The NPK levels (150 mg N/L; 75 mg P/L and 350 mg K/L) in the NS, which was regarded an intermediate fertilization scheme, showed a rise in nutritional value with high phenols, flavonoids and antioxidant activity in plants. *S. cypria* grown in N75 levels revealed a decreased plant fresh weight and chlorophylls content while plants grown in N300 levels revealed increases in mineral accumulation, nutrient and water use efficiency. The NPK and the K550 levels caused oxidative stress as demonstrated by the raised lipid peroxidation and the stimulation of enzymes’ antioxidant activities. The P50 levels in the NS, increased the plant biomass and water use efficiency (WUE) and revealed the lower oxidative stress (malondialdehyde) and increased enzymes antioxidant (superoxide dismutase and peroxidase) activities. As a result, modifying the NS composition in hydroponic culture for *S. cypria* by using P levels of 50 mg P/L, higher biomass, nutritive value and WUE can be obtained.

## 1 Introduction

Medicinal and aromatic plants (MAPs) have garnered increased attention recently [1,2]. Plants from Lamiaceae family especially, are of fascination as a source of natural bioactive components that may find use in the food, cosmetics, and pharmaceutical industries. The genus *Sideritis* L. is of global economic significance, with the plant biomass to be utilized as raw or dry material, and the infusions of mountain tea (*Sideritis* spp.) to give a present characteristic aroma [3,4]. However, *Sideritis* is characterized as oil-poor with low essential oil (EO) content [3] which limits its EO use in the industrial cosmetics and perfumery sector. Noteworthy, traditional medicine in Mediterranean region and thereafter in Cyprus, use *Sideritis* species to treat a variety of illness, including respiratory issues, stomach pain, flu and common colds [5]. This is related to the rich nutritional profile and phytochemical characteristics of the plant extracts and infusions that render *Sideritis* species of increased acceptance and use. The *Sideritis* species bio-properties includes but not limited to the anti-inflammatory, antibacterial, cytotoxic, and antioxidant properties [[6], [7], [8]]. *Sideritis perfoliata* L. subsp. *perfoliata* and *Sideritis cypria* Post are the two species that are commonly used in Cyprus, with the latter to be an endemic species and less commercialized or not at all. Both species have high similarities but also differences as it was indicated by the first comparative study between the two species from authors, highlighted the existence of iridoids and one methylated flavone in the *S. perfoliata* infusion compared to *S. cypria* [4].

The rising interest in researching MAPs and their properties is followed by the increased demands by the industry to receive continues, constant and of high-quality MAPs products. However, the lack of knowledge and the empirical horticulture practices applied by the growers, boost the demands for studies related to the sustainable cultivation practices and fertilization needs for MAPs in conjunction with the environmental and human safety [9]. In case of under-exploded species such as *S. cypria*, this is inevitable, as no studies on the crop needs for sustainable agriculture are evidenced.

Various soil types, microclimates, seasons, altitude, genetic material, and cultivation techniques, all affect the biochemical composition of MAPs [10]; with fertilization management to have a substantial role in the MAPs composition [11,12]. Along with other nutritional and metabolic characteristics, the mineral accumulation in plant tissue is highly intriguing and has a basic function in plant metabolism. Mineral support to cover the plant needs, is achieved by various methods, such as spray treatment, direct mineral use in soil or hydroponics, and control of mineral antagonism and accessibility [11,[13], [14], [15]]. Nevertheless, even when complying to environmental restrictions, overuse of synthetic fertilizers and incorrect usage of manure or organic materials enriched in minerals, especially heavy metals, can have a negative influence on crop performance, food quality, and environmental safety [16]. When paired with soil greenhouse conditions, hydroponics is a crop cultivation technology that is isolated from soil and the many constraints generated from crops produced in soil, allowing for the mineral intake management [17]. The last decades, increased research in soilless culture with MAPs is evidenced [14,18,19]. Therefore, research studies related to the tailoring nutrient solution (NS) needs [11,20,21], to the desired ammonium to nitrogen ratio in the NS [19,22], and to the role of ions to alleviate minerals antagonisms and oxidative stress [23,24] were implemented.

Nitrogen (N), potassium (K), and phosphorus (P) are three elements for crop development and have a crucial role in the physiology and plant growth [25]. All the three elements play a fundamental role on MAPs growth and biosynthesis of various components with medicinal properties [14,15,26]. Nitrogen is the most required mineral by the plant and can be provided in two forms, either ammonium or nitrate-nitrogen, with the latter to be more preferrable form by the plants [27]. Adequate N levels are fundamental for the biochemical metabolites (i.e. alkaloids, flavonoids, and EOs) which are responsible for the MAPs properties [28], whereas lower or excess N levels might negatively affect the crop yield and the metabolites synthesis. Potassium stimulates enzymes involved in photosynthesis, stomatal regulation, protein synthesis, and other metabolic processes, enhance plant resistance to abiotic and biotic stressors, hence boosting overall plant health and productivity [29] and impact the MAPs components quality [30]. Phosphorus is necessary for root growth and enhance the mineral and water absorption [31] but also contributing to plants secondary metabolisms [32] while P can antagonize N accumulation in plants [33].

The objectives of the current study were i) the successive establishment of an underexplored MAPs species i.e. *S. cypria*, in an intensive crop production system like hydroponics and ii) to investigate the mineral impacts when applied in different levels in the NS on plant growth, physiology, nutrient content, water and nutrient use efficacy when plants were grown under decreased or increased levels of N, K and P in hydroponics.

## 2 Materials and methods

### 2.1 Cropping system, plant material and growth conditions

The present study was implemented at Cyprus University of Technology, in Cyprus, in a plastic multi-span greenhouse with controlled climatic conditions. The cropping system adapted was the close hydroponic system, as the NS was recirculated throughout the cultivation period, well known as Nutrient Film Technique-NFT, with modifications to host the tested plant species, *S. cypria*. The hydroponic installation has been described previously [34]. In brief, 21 twin-white plastic NFT channels were aligned with 21 catchments tanks (60 L), to create 21 independent hydroponic units. Each unit was coupled with a separate water replenishment tank (60 L), whereas the volume (in litres) of the water consumed by the plants was recorded and sum up for each unit. Plants absorbed NS, which was replenished through automatic refill of water from the replenishment tank. Each hydroponic unit accompanied 12 plants, resulting in 36 plants per treatment, 252 plants in total.

Seven different NS were considered for study, by fluctuation of the three main macronutrients, nitrogen-N, phosphorus-P, and potassium-K, in three levels (in mg/L) as follows: i) NPK (150 mg N/L, 75 mg P/L and 350 mg K/L); ii) N75 (75 mg N/L, 75 mg P/L and 350 mg K/L); iii) N300 (300 mg N/L, 75 mg P/L and 350 mg K/L); iv) K150 (150 mg N/L, 75 mg P/L and 150 mg K/L); v) K550 (150 mg N/L, 75 mg P/L and 550 mg K/L); vi) P50 (150 mg N/L, 50 mg P/L and 350 mg K/L); and vii) P100 (150 mg N/L, 100 mg P/L and 350 mg K/L). Mean values for the air temperature and humidity in the greenhouse were 24.6^o^C and 51.3 %, respectively, during daytime.

*S. cypria* seedlings were prepared by the Department of Aromatic Plants, Ministry of Agriculture, Cyprus. Seedlings were placed into netted pots filled up with perlite, placed at the NFT channels, and were kept for 15 days with standard NS, to alleviate any possible transplanting stress and allowing root elongation. The standard NS composition used is presented in Table S1. After 15 days at the NFT system the plants were exposed to the modified NS for 44 days (Table S1). The modified NS were monitored daily and adjusted accordingly, with H_2_SO_4_ (5 % v/v) to reduce the pH to 5.8, and by adding relevant modified NSs to adjust the EC to 2.3 dS/m.

### 2.2 Plant growth

*S. cypri*a plants were grown for 44 days in seven modified NS in this study. At the end of the experiment, six plants from different replicates per treatment were selected and analysed for plant growth. Plant upper biomass fresh (g) and dry weight (g) were determined, and dry matter content was computed (%).

### 2.3 Plant physiology

Leaf relative chlorophyll content was monitored by using an optical chlorophyll meter (SPAD-502, Minolta, Osaka, Japan). Leaf chlorophyll fluorescence of PSII (Fv/Fm) was measured with the OptiSci OS-30p Chlorophyll Fluorometer (Opti-Sciences, Hertfordshire, UK). Photosynthetic leaf pigments were determined after extraction of fresh plant tissue (six replicates/treatment; each replicate was a pool of tissue from two plants). Following extraction and spectrophotometric measures, the chlorophyll *a* (Chl a), chlorophyll *b* (Chl b), total chlorophyll (total Chl) and carotenoids content were computed and results were expressed in mg per g of fresh weight [35,36]. Moreover, the Chla:Chlb and Carotenoids:Total Chls ratios were calculated.

### 2.4 Nutrient content in plant tissue, water and nutrient use efficiency

At the end of the experiment, the upper parts of the plant (leaves and stems) were sampled and further were used to determine the nutrient content, considering six replicates per treatment (replicate was a pool of three plants). Plant tissue was dried at 42 °C, then it was ash-burned at 470 °C for 6 h and then acid digested (2 M HCl). The extracts were used for macro- and micronutrients determination. Potassium (K), sodium (Na), and phosphorous (P) content was determined as reported in prior study [23]. Calcium (Ca), magnesium (Mg), iron (Fe), zinc (Zn), and copper (Cu) were determined by an atomic absorption spectrophotometer (PG Instruments AA500FG, Leicestershire, UK). Nitrogen was determined by the Kjeldahl method (BUCHI, Digest automat K-439 and Distillation Kjeldahl K-360, Flawil, Switzerland). Data were presented as g/kg and mg/kg of dry weight for macronutrients and micronutrients, respectively.

The quantity of water and nutrient solutions consumed by plants were monitored, by marked the adding of the modified stock solutions in the NS and the water from replenishment tanks, and subtracting the nutrients and water remained at the drainage solution, at the end of the cropping period. Water uptake (L/plant) was computed by recording the total quantity of water utilized by the plants during the cropping period. Nutrient uptake (mL/plant) was calculated.

Water use efficiency [37] was estimated by the ratio of the crop biomass produced and the water consumed, as described:

(1) $$\text{Water}\;\text{Use}\;\text{Efficiency}=\frac{\text{Biomass}\;\left(g\;\text{perplant}\right)}{\text{Water}\;\left(L\;\text{per}\;\text{plant}\right)}$$

Nutrient use efficiency [38] was estimated by the ratio of crop biomass produced and the volume of the NS supply, as described:

(2) $$\text{Nutrient}\;\text{Use}\;\text{Efficiency}=\frac{\text{Biomass}\;\left(g\;\text{per}\;\text{plant}\right)}{\text{NS}\;\left(\text{mL}\;\text{per}\;\text{plant}\right)}$$

### 2.5 Total phenols, total flavonoids, and antioxidant capacity

Methanolic extracts of the plant tissue (six replicates/treatment) were used for total phenols and flavonoids content determination. The total antioxidant activity was assessed by three different assays, as the 2,2-diphenyl-1-picrylhydrazyl (DPPH), the ferric reducing antioxidant power (FRAP) and the 2,2′-azino-bis(3-ethylbenzothiazoline-6-sulphonic acid (ABTS) assay.

Total phenols content of leaf extract was determined at 755 nm by using the Folin–Ciocalteu method, following the chemical reactions [11]. Results were presented in gallic acid equivalents (mg GA/g of fresh weight-FW). Total flavonoids content was determined at 510 nm by employing the aluminium chloride colorimetric method [39] following modifications [11]. Total flavonoid content was computed as rutin equivalents (mg rutin/g FW).

Free radical-scavenging activity of the leaf extracts was measured at 517 nm for DPPH and at 593 nm for FRAP [40]. The ABTS assay was implemented based on the methodology described by Woidjylo et al. [41]. Results were expressed as Trolox ((±)-6-Hydroxy-2,5,7,8-tetramethylchromane-2-carboxylic acid) equivalents (mg trolox/g FW).

### 2.6 Lipid peroxidation, hydrogen peroxide, and enzymes antioxidant activity

Hydrogen peroxide (H_2_O_2_) content was determined at 390 nm according to Loreto and Velikova [42]. Six samples (two pooled plants/sample) were used as replicates for each treatment and the results were expressed as μmol H_2_O_2_/g fresh weight.

Lipid peroxidation, in terms of malondialdeyde content (MDA), was determined according to De Azevedo Neto et al. [43]. The absorbance was recorded at 532 nm and corrected for non-specific absorbance at 600 nm. Results were expressed as nmol of MDA/g fresh weight.

The antioxidant enzymes activity, such as superoxide dismutase (SOD) and catalase (CAT) were determined by measuring the absorbance at 560 nm and at 240 nm, respectively [14]. Peroxidase activity (POD) was determined following the increase in absorbance at 430 nm [34]. Results were presented as enzyme units/mg of protein. The protein content in leaf tissue was measured using the Bradford method.

### 2.7 Statistical analysis

Data were tested for normality and statistically analysed with the IBM SPSS v.22 (IBM Corp., Armonk, NY, USA) and results were expressed as means value ± standard error (SE). Differences between mean values were compared using ANOVA, followed by Duncan's Multiple Range Test (DMRT) at P < 0.05. The correlation coefficients between N, P and K levels in the NS with individual parameters tested by Pearson's correlation test.

## 3 Results

*S. cypria* plants grown under NFT system revealed EC between 1.98 and 2.43 dS/m, with higher values for K550 NS treatment (Fig. S1). Thirteen days after the application of the modified NS, the EC of the NSs was boosted to 2.3 dS/m based on the increased plant development and mineral needs until the end of the study. A consistent pattern of pH variation was detected among the several modified NS.

Table 1 shows the impacts of the different NS applications on *S. cypria* biomass production. The N75 use in the NS significantly reduced biomass fresh weight, when compared to all the examined treatment including the NPK treatment (as considered an intermediate-control). The highest biomass fresh weight was produced by plants using the P50 in the NS, presenting an increase of up to 179.2 % compared to NPK treatment, while the same treatment produced plants with the lowest biomass dry matter content. Similarly, the N75 in the NS decreased biomass dry weight compared with K150, K550 and P50, but also had the highest biomass dry matter content.

**Table 1:** Effect of nitrogen (N: 75, 150, and 300 mg/L), potassium (K: 150, 350, and 550 mg/L), and phosphorus (P: 50, 75, and 100 mg/L) in the nutrient solution on *Sideritis cypria* upper fresh weight (FW; g/plant), and dry weight (g/plant) and dry matter (DM; %) in plants grown hydroponically in NFT. Significant differences (*P* < 0.05) among modified NS are marked by different letters.

Nutrient solution	Biomass FW	Biomass DW	Biomass DM
NPK (150-75-350)	53.08 ± 6.23c	8.01 ± 1.03 ab	15.01 ± 0.66b
N75	30.38 ± 5.19d	6.08 ± 0.33b	17.21 ± 0.35a
N300	64.30 ± 7.35bc	8.16 ± 0.94 ab	12.70 ± 0.17cd
K150	70.10 ± 3.68abc	9.68 ± 0.51a	13.82 ± 0.07bc
K550	73.13 ± 6.12 ab	9.50 ± 0.79a	13.00 ± 0.63cd
P50	84.83 ± 5.22a	9.80 ± 0.59a	11.57 ± 0.24d
P100	63.72 ± 6.68bc	8.23 ± 0.90 ab	12.89 ± 0.42cd

No differences were found on the relative chlorophyll content and chlorophyll fluorescence (Table 2). K150 and P100 nutrient solutions increased *S. cypria* chlorophyll *a* content, compared to plants grown with N75 and P50. Furthermore, the P100 nutrient solution produced plants with the highest chlorophyll *b* and total chlorophylls content, an increase of 89.3 % and 40.5 % respectively, over plants grown with the N75 nutrient solution, which had the lowest values. However, the N75 treatment produced plants with the highest carotenoids content, and subsequently the highest carotenoids to chlorophylls ratio, as well as the highest Chl a:Chl b ratio. No differences were found between leaf chlorophyll fluorescence (averaged in Fv/Fm of 0.81) and SPAD values (averaged in 51.12) among the plants grown in the tested modified NSs.

**Table 2:** Effect of nitrogen (N: 75, 150, and 300 mg/L), potassium (K: 150, 350, and 550 mg/L), and phosphorus (P: 50, 75, and 100 mg/L) in the nutrient solution on *Sideritis cypria* SPAD value, leaf chlorophyll fluorescence (Fv/Fm), chlorophyll content (a, b, total; mg/g fresh weight), carotenoids content (mg/g fresh weight), Chla:Chlb and Carotenoids:Total Chls ratios, in plants grown hydroponically in NFT. Significant differences (*P* < 0.05) among modified NS are marked by different letters.

Nutrient solution	SPAD	Chlorophyll fluorescence	Chlorophyll *a*	Chlorophyll *b*	Total Chlorophylls	Carotenoids	Chlorophyll *a*/Chlorophyll *b*	Carotenoids/Total Chlorophylls
NPK (150-75-350)	52.55 ± 4.19	0.80 ± 0.011	1.09 ± 0.02 ab	0.42 ± 0.05 ab	1.51 ± 0.06bcd	0.23 ± 0.020bc	2.72 ± 0.41 ab	0.15 ± 0.018bc
N75	46.42 ± 1.47	0.81 ± 0.007	0.97 ± 0.07b	0.28 ± 0.03c	1.26 ± 0.06d	0.28 ± 0.009a	3.55 ± 0.49a	0.22 ± 0.003a
N300	50.17 ± 5.42	0.83 ± 0.002	1.10 ± 0.10 ab	0.45 ± 0.07 ab	1.56 ± 0.17abc	0.21 ± 0.012c	2.49 ± 0.21b	0.14 ± 0.012c
K150	55.52 ± 4.63	0.81 ± 0.009	1.23 ± 0.02a	0.48 ± 0.02 ab	1.71 ± 0.04 ab	0.25 ± 0.003abc	2.56 ± 0.08b	0.15 ± 0.006bc
K550	55.43 ± 2.04	0.80 ± 0.012	1.08 ± 0.04 ab	0.38 ± 0.03bc	1.46 ± 0.05bcd	0.26 ± 0.022 ab	2.89 ± 0.30 ab	0.18 ± 0.017b
P50	51.97 ± 2.11	0.80 ± 0.004	1.05 ± 0.04b	0.38 ± 0.02bc	1.43 ± 0.06cd	0.23 ± 0.010bc	2.82 ± 0.07 ab	0.16 ± 0.000bc
P100	45.85 ± 3.98	0.80 ± 0.033	1.24 ± 0.01a	0.53 ± 0.02a	1.77 ± 0.01a	0.24 ± 0.003abc	2.32 ± 0.09b	0.14 ± 0.003c

**Fig. 1:**
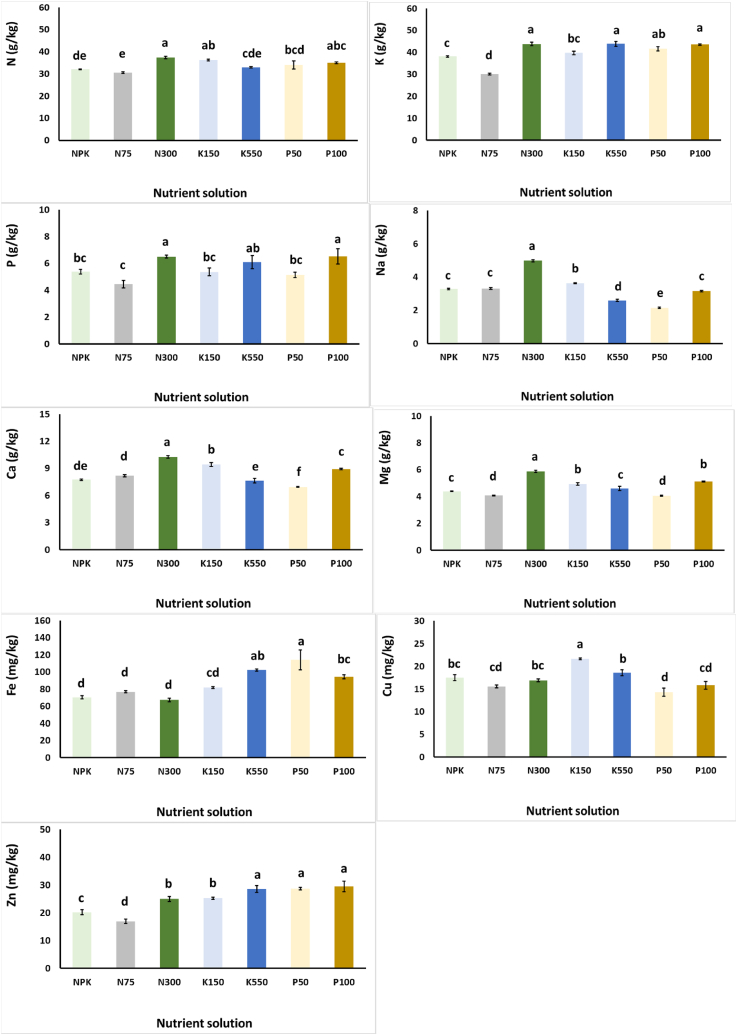
Effect of nitrogen (N: 75, 150, and 300 mg/L), potassium (K: 150, 350, and 550 mg/L), and phosphorus (P: 50, 75, and 100 mg/L) in the nutrient solution on *Sideritis cypria* macronutrients and micronutrients content, in plants grown hydroponically in NFT. The NPK is considered as the mid-levels for N150, P75 and K350. Significant differences (*P* < 0.05) among modified NS are marked by different letters.

Fig. 1 exhibits the effects of the different NS used for the growth of *S. cypria* plants, on plants mineral content. Compared to the control (NPK) treatment, the N300, K150 and P100 application increased the N content of leaves. Similarly, the N75 nutrient solution also decreased the K content of plant leaves, compared to the control treatment. Phosphorus accumulated more in plants grown in N300, and P100 treatments. Furthermore, the highest Na, Ca and Mg content was exhibited in the leaves when plants grown using the N300 treatment, whereas the K550 and P50 treatments reduced the Na content, and the P50 treatment reduced Ca and Mg content, as to the control NS. Micronutrients were also affected by the examined NSs. The highest Fe content was observed in the leaves of plants grown with the P50 in the NS, while a significant decrease in leaf Zn was found in the N75 treatment, compared to NPK. Finally, the highest Cu content was observed in the leaves of plants grown with K150 in the NS.

**Fig. 2:**
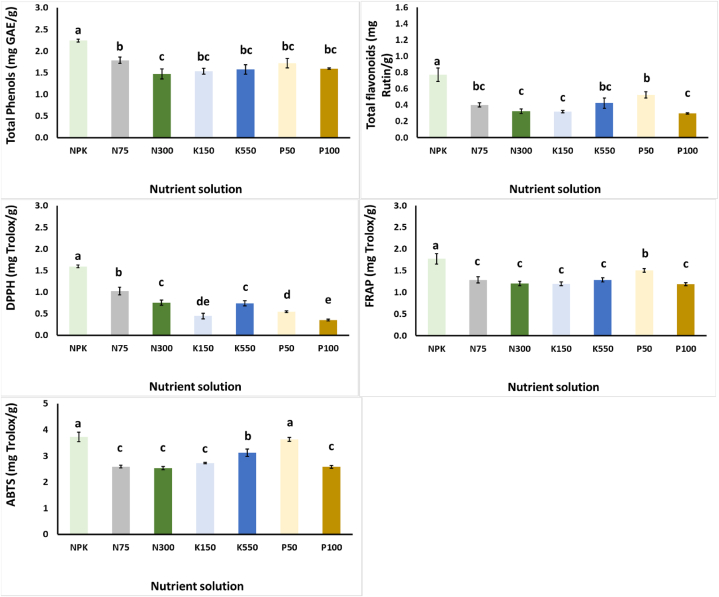
Effect of nitrogen (N: 75, 150, and 300 mg/L), potassium (K: 150, 350, and 550 mg/L), and phosphorus (P: 50, 75, and 100 mg/L) in the nutrient solution on *Sideritis cypria* on total phenols (mg GA/g Fw), antioxidant activity (DPPH, FRAP, ABTS; mg Trolox/g Fw), and total flavonoids (mg Rutin/g Fw) in plants grown hydroponically in NFT. The NPK is considered as the mid-levels for N150, P75 and K350. Significant differences (*P* < 0.05) among modified NS are marked by different letters.

**Fig. 3:**
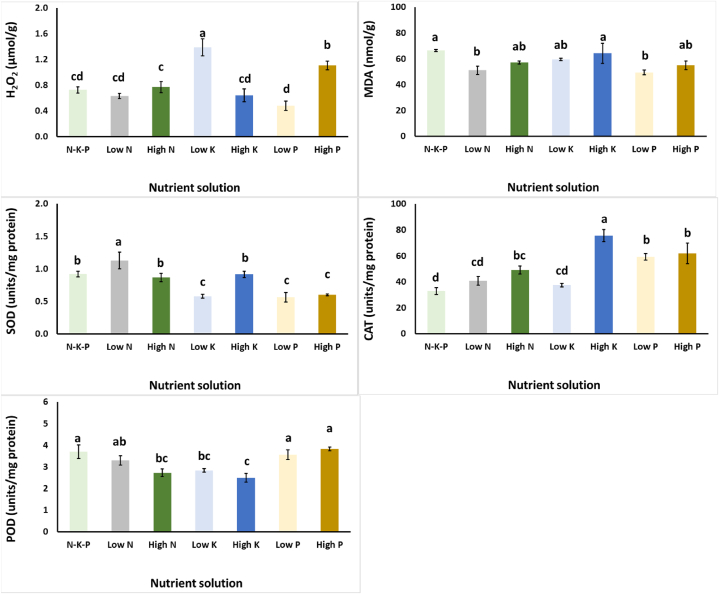
Effect of nitrogen (N: 75, 150, and 300 mg/L), potassium (K: 150, 350, and 550 mg/L), and phosphorus (P: 50, 75, and 100 mg/L) in the nutrient solution on *Sideritis cypria* on hydrogen peroxide- H_2_O_2_ (μmol/g), lipid peroxidation-MDA (nmol/g) and antioxidant enzymes activity of superoxide dismutase (SOD; units/mg protein), catalase (CAT; units/mg protein), and peroxidase (POD; units/mg protein), in plants grown hydroponically in NFT. The NPK is considered as the mid-levels for N150, P75 and K350. Significant differences (*P* < 0.05) among modified NS are marked by different letters.

All modified NS treatments decreased the content of the total phenols and the total flavonoids of *S. cypria* plants over the NPK treatment, although a decrease in flavonoids was observed with the application of P50 over the P100 treatment (Fig. 2). A similar result was obtained for the antioxidant capacity of plants, as assayed by DPPH and FRAP, although the ABTS of plants grown with P50 did not differ significantly from the control treatment. Even though total phenolics and antioxidants generally decreased with the different variations in the NS, in comparison to the control treatment, *S. cypria* plants that grown in N75 and P50 in the NS, revealed less oxidative stress due to decreased levels of MDA levels (Fig. 3). The N75 treatment increased the H_2_O_2_ levels over the control treatment and this reflected the increased SOD levels too. Finally, CAT activity was the highest with the application of K550, while POD was decreased with the application of N300, K150 and K550 compared to the NPK treatment (Fig. 3).

The WUE and NUE as affected by the different NS is presented in Table 3. The highest NUE was observed with the N300 treatment while the least NUE was found with the N75 and K150 treatments. Increased WUE was found with the N300 and P50 treatments.

**Table 3:** Effect of nitrogen (N: 75, 150, and 300 mg/L), potassium (K: 150, 350, and 550 mg/L), and phosphorus (P: 50, 75, and 100 mg/L) in the nutrient solution on nutrient uptake (mL/plant), water uptake (L/plant), nutrient use efficiency (NUE: g DW/mL of NS per plant), and water use efficiency (WUE: g DW/L H_2_O per plant) of *Sideritis cypria* plants grown hydroponically in NFT. Significant differences (*P* < 0.05) among modified NS are marked by different letters.

Nutrient solution	Nutrient uptake	Water uptake	Nutrient use efficiency	Water use efficiency
NPK (150-75-350)	22.51 ± 0.69b	7.62 ± 0.23b	0.337 ± 0.013d	0.989 ± 0.031c
N75	21.59 ± 0.55b	7.83 ± 0.20 ab	0.265 ± 0.004e	0.739 ± 0.020d
N300	12.95 ± 0.26e	6.09 ± 0.12d	0.606 ± 0.013a	1.285 ± 0.024a
K150	34.82 ± 0.89a	8.33 ± 0.21a	0.263 ± 0.006e	1.103 ± 0.027b
K550	15.67 ± 0.40d	8.00 ± 0.20 ab	0.576 ± 0.014 ab	1.129 ± 0.030b
P50	17.06 ± 0.43cd	6.95 ± 0.18c	0.546 ± 0.014b	1.338 ± 0.032a
P100	18.48 ± 0.38c	6.93 ± 0.14c	0.429 ± 0.011c	1.138 ± 0.021b

Linear correlation coefficients were calculated to demonstrate the contribution of the N (Table S2), K (Table S3) and P (Table S4) concentrations in the NS for each individual factor. Regarding N levels in the NS, there was a positive correlation with N (75-150-300 mg/L) concentrations and leaf minerals content (N, Na, K, Mg, Ca, P, and Zn), and biomass fresh weight, and a negative correlation with leaf Fe content, dry matter content, total carotenoids and ratio of total carotenoids/total chlorophylls (Table S2). Regarding K levels in the NS, there was a positive correlation with K (150-350-550 mg/L) concentrations and SOD and CAT enzymes activities, and a negative correlation with chlorophyll *a* and total chlorophylls, and leaf N, leaf Na and leaf Ca content (Table S3). Regarding P levels in the NS, there was a positive correlation with P (50-75-100 mg/L) concentrations and leaf Na, leaf Mg, leaf Ca, and leaf P content, and chlorophylls (Chl a, Chl b, total Chls) content, and a negative correlation with ABTS (Table S4).

## 4 Discussion

Natural sources account for 50 % of contemporary pharmaceuticals in general, with increasing demands for MAPs production and use in different industrial and culinary sectors [44]. However, the excessive MAP harvesting in the wild increases their risk of extinction, especially endangered species [45]. Growers in many cases are not aware of the appropriate and sustainable cultivation practices for MAPs, which discourage them to continue activities on MAP sector and/or produce fresh or dry material of undesirable quantity and properties. Efforts are underway to produce MAPs for a consistent supply and support the industry. In the present research, the N, P and K levels in the NS in hydroponically grown *S. cypria* were examined, as the appropriate supply of essential elements for plant growth, physiology and biochemical components is important for MAPs properties [46].

In the present study, the lower P levels (i.e. 50 mg P/L) in the NS, increased (up to 59.8 %) the plant biomass compared to the NPK treatment (which considered as common NS composition) and the higher P levels. In contrast, lavender (*Lavandula angustifolia* Mill.) plants grown in perlite with different P levels (30–70 mg/L) had increased plant biomass at the high P levels of 70 mg/L in the NS [11]. Spearmint (*Mentha spicata*) plants grown in deep flow technique-DFT under different P levels (30–70 mg/L) had similar plant biomass production while the high P levels of 70 mg/L in the NS impacted positively the spearmint metabolism and EO properties [47]. Those differences on the P impacts on plant biomass is related to the plant species and hydroponic system used in different studies, indicating the importance of tailoring the NS composition for different crops. The lower N levels (i.e. 75 mg N/L) resulted to decreased biomass fresh weight but increased dry matter content. Therefore, plants benefited to the mid N levels (150 mg/L) compared to the lower N (75 mg/L), despite the fact that *S. cypria* seeds used to produce the studied plants originated from wild ecotypes in the past and never breeded or selected for high growth rates in regards to high N supply, rather than their natural evolution throughout the years. Obviously, plants had to adapt to semi-arid climatic condition of the Mediterranean region, with poor vegetation. Decreased *Salvia officinallis* L. fresh biomass was also reported in plants grown under low levels of N (i.e. 140 mg N/L) in the NS being in accordance with the present outcomes [48]. Lavender plants grown in N ranged from 150 to 250 mg/L in the NS had the same biomass [11], being in agreement with the present outcomes, as *S. cypria* grown in 150 and 300 mg/L of N in the NS revealed the same fresh biomass. *S. cypria* is mainly purchased in the market as dry material, compared to raw-fresh material. Therefore, the plants grown in low N had the lowest dry weight, which is considered as unfavourable NS application for the *S. cypria* production. All the other NS application had similar high dry biomass production with the greatest values to be observed in plants grown in lower P treatment. Plant biomass production should not be of priority for growers without considering the maintenance and/or improve the bioactive properties of MAPs.

The increased P levels in the NS revealed high chlorophylls (a, b and total**)** content but low ratios of Chla:Chlb and Carotenoids:Total Chlorophylls, showing the preservation and/or improvement of leaf photosynthetic capability and plant growth [49], as Chl b changes to Chl a through the chlorophylls degradation [50,51]. Khammar et al. [48] reported that the increased N levels (up to 200 mg N/L) in the NS resulted in higher content of chlorophylls and carotenoids, which is partially true in the present study, as total chlorophylls content was higher in plants grown at N300 than at N75, but the maximum content was found in case of P100 indicating the important role of other minerals for good plant performance as well as the appropriate balance among the different minerals in the NS.

The K and N (in the form of nitrate) uptake are positively correlated in plants, as stated in prior reports [52], being in alignment with the present outcomes as increased K content was found in plants grown under increased N and P, and obviously under increased K concentration in the NS. Nitrogen accumulation in leaves was high at N300 application, but decreased with the N75 and the K550 application in the NS, as previously reported in spearmint plants grown in high K levels in the NS, in hydroponics [14]. Nitrogen levels in the NS were positively correlated (*p* < 0.01) with the accumulation of P in plant tissue while previous reports indicated that N uptake might be reduced in case of P shortage [53], providing evidence that even the lower P levels (P50) used in the current research should not be considered as ‘’P shortage’’ rather than the adequate P levels for the plant needs. Obviously, a study with a narrow range of P levels could provide more insides for the optimum level of P for the tested species, as successfully implemented for spearmint [47] and lavender [11], previously. Higher concentrations of N, P, and K in the NS led to mineral buildup in plant tissue, illustrating the efficacy of biofortification procedures used in agriculture today, and providing products of high nutritive value on minerals. In fact, accumulate nutrients in plant tissue must be associated with the production of biomass and desirable biochemical attributes of the products, such as phenols and flavonoids. However, this is not evidenced at the present study, as neither biomass production was related to the high N, P and K levels nor the phenols and flavonoids content were mirrored to the high N, P and K levels applied through the NS.

The highest NUE observed with the N300 treatment, which is a combination of the low nutrient uptake, and the relevant biomass produced by the plants grown under the high N levels. However, plant biomass produced at N300 application was not the maximum one, compared to the biomass produced under the high P application (P100) in the NS, indicating that part of the N was used on other metabolic process, rather than for biomass production [33]. In general, the sustainable and appropriate utilization of N applied through fertilization is of great importance for environment and living organisms, as more than 50 % of applied N fertilizers are lost in the environment with severe problems occurred [54]. The highest WUE was observed at the N300 and P50 treatments. The high WUE is extremely important, particularly in regions with water deficits, such as the Mediterranean region and countries, including Cyprus. In that sense, applications of N75 or NPK are not recommended due to their low WUE and NUE (including the K150 application) values.

In cropping systems, plants are generally subjected to one or a series of biotic and abiotic stresses that may considerably influence plant growth, yield and quality performances [12,55,56]. In recent decades, research is now focusing on abiotic stressors such as excessive salinity, harsh temperatures, drought, nutrient shortages, and soil pollution [23,55,57]. Plants when exposed to stress conditions, activate non-enzymatic and enzymatic antioxidant mechanisms to scavenge the produced reactive oxygen species (ROS) [23]. Potassium has a role in multiple signalling systems that protect plants against stress and activate antioxidant defence mechanisms [58]. In the present work, increased MDA levels, as regular plant stress indicator, were observed at NPK (150 mg N/L; 75 mg P/L and 350 mg K/L) and K550 treatments. In this case, the K excess stimulated oxidative stress, rather than alleviated the referred stress. In oppose, at low N (N75) and low P (P50), MDA had low levels, which combined with the activation of different enzymes antioxidants (SOD, POD) that took place. Spearmint plants grown in DFT hydroponic system, with various K concentration in the NS (275–375 mg/L) stimulated the enzymatic antioxidant activities (SOD, CAT, ascorbate peroxidase-APX) while high K applications boosted the total phenols content and antioxidant capacity (DPPH, FRAP) [14]. However, in the present study, the use of the K550 in NS reduced the total phenolics and flavonoids, and this difference might be related to the fact that the K high levels are different among the two studies (550 mg/L and 375 mg/L at Chrysargyris et al. [14]) as well as the tested plant species. Noteworthy to mention that the higher phenols content, the higher flavonoids content and raised antioxidant capacity, were observed in plants that grown at NPK (medium levels) applications, which reflected the high nutritive value of the examined species. Several studies indicated the positive correlation on the total phenolics and flavonoids with the high total antioxidant capacity of the plant, whereas in other did not reveal any relationship [59].

There has been an explosion in research interest in studies on MAPs and tailoring factors that include enhanced biomass, higher WUE and NUE, biofortification methods with minerals and production of MAPs with high biocidal capabilities [14,47]. Both the nutrient concentration and the nutrient ratio (i.e. N/K) should be constantly tracked and adjusted for each plant species. Farmers may promote the growth, production, and synthesis of valuable molecules, as well as the overall efficacy of MAP products, by knowing their specific fertilization needs and adjusting nutrient levels, accordingly, providing a well-balanced fertigation scheme to the plants. Hydroponics is an effective cultivation practice to that direction by using a regulated fertigation system [17]. However very few studies on MAPs grown in hydroponics considered the cost of production, and therefore, not every MAP has the potential to be economically viable under hydroponic cultivation scheme in the current market [60]. Production of biomass with high added value as well saving fertilizers and water (with the high WUE and NUE) is supporting the economic production of MAPs. The worldwide market for MAPs is expected to grow from USD 800 million to $50 trillion by 2050, with increased demands for sustainable production of MAP and applied technology on agriculture sector [61].

## 5 Conclusions

In the present study, the effects of N, P and K levels in the NS on the growth, mineral content and biochemical characteristics, of *S. cypria* plants grown in hydroponics were examined. The NPK levels (150 mg N/L; 75 mg P/L and 350 mg K/L) in the NS, considered as an intermediate fertilization scheme, revealed increased nutritive value with high phenols, flavonoids and antioxidant capacity in plants. The low N levels (75 mg N/L) in the NS, decreased plant fresh weight and chlorophylls content, but increased dry matter content. The increased N levels to 300 mg/L in the NS, increased mineral accumulation, increased WUE and NUE but decreased the total phenols, total flavonoids and nutrient and water uptake. The low K levels (i.e. 150 mg K/L) in the NS, increased Cu content in plant tissue. The high K levels (i.e. 550 mg K/L) in the NS decreased the total phenols and total flavonoids content but raised the K accumulation in plants. The high P levels (i.e. 100 mg P/L) in the NS stimulated the chlorophylls content and K and P accumulation. The low P levels (i.e. 50 mg P/L) in the NS, increased the plant biomass, both in fresh and dry weight as well as WUE. The MDA levels were increased at NPK and high K treatments, while in case of low N and low P, various enzymes antioxidants (SOD, POD) were stimulated to scavenge oxidative stressors. The increased levels of N, P, K in the NS resulted in nutrient accumulation in the plant tissue. The cultivation of MAPs using controlled culture strategies, such as hydroponics systems, may supply fresh and dry biomass of the desirable quality and composition, which are needed by industries. Further study is required to investigate the appropriate levels for Ca and Mg, as well as various micronutrients like as Cu, Zn, Fe, and manganese (Mn), which are involved on plant metabolic processes.

## CRediT authorship contribution statement

**Antonios Chrysargyris:** Writing – review & editing, Writing – original draft, Visualization, Validation, Supervision, Software, Resources, Project administration, Methodology, Investigation, Formal analysis, Data curation, Conceptualization. **Nikolaos Tzortzakis:** Writing – review & editing, Writing – original draft, Visualization, Supervision, Resources, Project administration, Methodology, Investigation, Funding acquisition, Formal analysis, Conceptualization.

## Data availability

Data will be made available on request.

## Funding

This research was funded by the Project ‘’Opti-AromaQ’’ EXCELLENCE/0421/0299 which is co-financed by the 10.13039/501100000780European Union and the Republic of Cyprus through the Research and Innovation Foundation.

## Declaration of competing interest

The authors declare the following financial interests/personal relationships which may be considered as potential competing interests: Antonios Chrysargyris is Associate Editor for the Heliyon Journal -AX. If there are other authors, they declare that they have no known competing financial interests or personal relationships that could have appeared to influence the work reported in this paper.
