# Supplementary material for: Nitrogen, phosphorus, and potassium requirements to improve Sideritis cypria growth, nutrient and water use efficiency in hydroponic cultivation
Source: Heliyon. 2024 Dec 4;11(1):e40755. doi: 10.1016/j.heliyon.2024.e40755 (PMC11699360; doi:10.1016/j.heliyon.2024.e40755)
Supplement: Multimedia component 1 [file mmc1.docx]

**Table S1.** Electrical conductivity (EC) and nutrient concentrations in the nutrient solution (NS) supplied to *S. cypria* plants grown in a recirculated hydroponic system.

| Parameters | S_tarter_ NS | ΝPK  (150-75-350) | Ν75 | Ν300 | K150 | K550 | P50 | P100 |
| --- | --- | --- | --- | --- | --- | --- | --- | --- |
| EC dS/m | 1.75 | 2.57 | 2.57 | 2.57 | 2.57 | 2.57 | 2.57 | 2.57 |
| K^+^ mmol/L | 7.55 | 8.95 | 8.95 | 8.95 | 3.84 | 14.06 | 8.95 | 8.95 |
| Ca^2+^ mmol/L | 3.50 | 3.74 | 3.74 | 3.74 | 3.74 | 3.74 | 3.74 | 3.74 |
| Mg^2+^ mmol/L | 1.00 | 2.88 | 2.88 | 2.88 | 2.88 | 2.88 | 2.88 | 2.88 |
| NH_4_^+^ mmol/L | 0.5 | 0.62 | 0.62 | 0.62 | 0.62 | 0.62 | 0.62 | 0.62 |
| NO_3_^-^ mmol/L | 13.72 | 10.71 | 5.35 | 21.42 | 10.71 | 10.71 | 10.71 | 10.71 |
| SO_4_^2-^ mmol/L | 1.29 | 1.56 | 1.56 | 1.56 | 1.56 | 1.56 | 1.56 | 1.56 |
| H_2_PO_4_^-^ mmol/L | 1.80 | 2.42 | 2.42 | 2.42 | 2.42 | 2.42 | 1.63 | 3.23 |
| Cl^-^ mmol/L | 1.49 | 1.49 | 1.49 | 1.49 | 1.49 | 1.49 | 1.49 | 1.49 |
| Fe μmol/L | 30.00 | 71.56 | 71.56 | 71.56 | 71.56 | 71.56 | 71.56 | 71.56 |
| Mn μmol/L | 5.00 | 18.21 | 18.21 | 18.21 | 18.21 | 18.21 | 18.21 | 18.21 |
| Zn μmol/L | 4.00 | 1.53 | 1.53 | 1.53 | 1.53 | 1.53 | 1.53 | 1.53 |
| Cu μmol/L | 1.00 | 4.72 | 4.72 | 4.72 | 4.72 | 4.72 | 4.72 | 4.72 |
| B μmol/L | 30.00 | 18.52 | 18.52 | 18.52 | 18.52 | 18.52 | 18.52 | 18.52 |
| Mo μmol/L | 0.50 | 0.52 | 0.52 | 0.52 | 0.52 | 0.52 | 0.52 | 0.52 |
|  |  |  |  |  |  |  |  |  |
| N/K |  | 1.27 | 0.64 | 2.53 | 2.95 | 0.81 | 1.27 | 1.27 |
| K/Total N |  | 0.79 | 1.57 | 0.39 | 0.34 | 1.24 | 0.79 | 0.79 |
| K/(K+Ca+Mg) |  | 0.40 | 0.40 | 0.40 | 0.22 | 0.52 | 0.40 | 0.40 |
| Ca/(K+Ca+Mg) |  | 0.34 | 0.34 | 0.34 | 0.44 | 0.27 | 0.34 | 0.34 |
| Mg/(K+Ca+Mg) |  | 0.26 | 0.26 | 0.26 | 0.34 | 0.21 | 0.26 | 0.26 |

Starter NS was introduced into the closed system for 15 days, before the application of the modified NS.
