# Supplementary material for: Nitrogen, phosphorus, and potassium requirements to improve Sideritis cypria growth, nutrient and water use efficiency in hydroponic cultivation
Source: Heliyon. 2024 Dec 4;11(1):e40755. doi: 10.1016/j.heliyon.2024.e40755 (PMC11699360; doi:10.1016/j.heliyon.2024.e40755)
Supplement: Multimedia component 3 [file mmc3.docx]

**Table S3.** Correlations coefficients and (p-values) between the K concentrations in the NS and the *Sideritis cypria* plant mineral content, growth, and physiology attributes.

|  | | **K levels in NS** | **N** | **Na** | **K** | **Mg** | **Ca** | **P** | **Fe** | **Zn** | **Cu** |  |
| --- | --- | --- | --- | --- | --- | --- | --- | --- | --- | --- | --- | --- |
| **K levels in NS** | **r** | 1 | -0.722* | -0.970** | 0.631 | -0.512 | -0.845** | 0.499 | 0.682 | 0.370 | -0.650 |  |
|  | **p** |  | 0.028 | 0.000 | 0.069 | 0.159 | 0.004 | 0.172 | 0.062 | 0.327 | 0.058 |  |
| **SPAD** | **r** | -0.004 | 0.108 | -0.069 | -0.066 | -0.009 | -0.021 | -0.023 | 0.058 | -0.037 | 0.005 |  |
|  | **p** | 0.988 | 0.276 | 0.483 | 0.503 | 0.926 | 0.835 | 0.816 | 0.560 | 0.710 | 0.959 |  |
| **Fv/Fm** | **r** | -0.424 | 0.074 | 0.198 | 0.136 | 0.246* | 0.223 | 0.058 | 0.013 | -0.090 | -0.011 |  |
|  | **p** | 0.402 | 0.541 | 0.100 | 0.261 | 0.040 | 0.063 | 0.634 | 0.915 | 0.457 | 0.929 |  |
| **FW** | **r** | 0.082 | 0.377** | -0.204* | 0.580** | 0.417** | 0.079 | 0.378** | 0.403** | 0.271** | 0.205* |  |
|  | **p** | 0.746 | 0.000 | 0.037 | 0.000 | 0.000 | 0.422 | 0.000 | 0.000 | 0.005 | 0.037 |  |
| **DW** | **r** | -0.038 | 0.279** | -0.153 | 0.455** | 0.337** | 0.071 | 0.278** | 0.324** | 0.194* | 0.184 |  |
|  | **p** | 0.880 | 0.004 | 0.120 | 0.000 | 0.000 | 0.470 | 0.004 | 0.001 | 0.047 | 0.062 |  |
| **DM %** | **r** | -0.343 | -0.635** | 0.223 | -0.811** | -0.564** | -0.063 | -0.544** | -0.466** | -0.538** | -0.216 |  |
|  | **p** | 0.506 | 0.000 | 0.063 | 0.000 | 0.000 | 0.603 | 0.000 | 0.000 | 0.000 | 0.075 |  |
| **Chl a** | **r** | -0.759* | 0.334** | 0.288** | 0.142 | 0.378** | 0.320** | 0.117 | -0.074 | -0.154 | 0.211* |  |
|  | **p** | 0.018 | 0.001 | 0.003 | 0.149 | 0.000 | 0.001 | 0.236 | 0.455 | 0.116 | 0.031 |  |
| **Chl b** | **r** | -0.603 | 0.275** | 0.193* | 0.300** | 0.431** | 0.279** | 0.215* | 0.042 | -0.058 | 0.155 |  |
|  | **p** | 0.085 | 0.005 | 0.048 | 0.002 | 0.000 | 0.004 | 0.028 | 0.671 | 0.559 | 0.116 |  |
| **Total Chls** | **r** | -0.790* | 0.330** | 0.268** | 0.213* | 0.423** | 0.325** | 0.161 | -0.034 | -0.130 | 0.204* |  |
|  | **p** | 0.011 | 0.001 | 0.006 | 0.029 | 0.000 | 0.001 | 0.100 | 0.732 | 0.185 | 0.037 |  |
| **Tot carotenoids** | **r** | 0.049 | 0.091 | 0.192* | -0.183 | 0.047 | 0.165 | -0.101 | -0.204* | -0.293** | 0.195* |  |
|  | **p** | 0.901 | 0.358 | 0.050 | 0.061 | 0.637 | 0.092 | 0.306 | 0.039 | 0.002 | 0.047 |  |
| **Chla/Chlb** | **r** | 0.304 | -0.022 | 0.055 | -0.256** | -0.201* | -0.040 | -0.181 | -0.150 | -0.041 | -0.043 |  |
|  | **p** | 0.427 | 0.824 | 0.576 | 0.009 | 0.039 | 0.684 | 0.064 | 0.131 | 0.680 | 0.667 |  |
| **Carot/totalChl** | **r** | 0.446 | -0.223* | -0.006 | -0.432** | -0.338** | -0.107 | -0.262** | -0.209* | -0.236* | 0.033 |  |
|  | **p** | 0.229 | 0.023 | 0.952 | 0.000 | 0.000 | 0.276 | 0.007 | 0.034 | 0.015 | 0.740 |  |
| **Phenols** | **r** | 0.044 | -0.288** | -0.188 | -0.204* | -0.295** | -0.219* | -0.072 | -0.003 | -0.156 | -0.206* |  |
|  | **p** | 0.911 | 0.003 | 0.055 | 0.037 | 0.002 | 0.025 | 0.467 | 0.972 | 0.111 | 0.036 |  |
| **DPPH** | **r** | 0.240 | -0.478** | 0.076 | -0.516** | -0.519** | -0.213* | -0.396** | -0.473** | -0.504** | -0.280** |  |
|  | **p** | 0.533 | 0.000 | 0.443 | 0.000 | 0.000 | 0.029 | 0.000 | 0.000 | 0.000 | 0.004 |  |
| **FRAP** | **r** | 0.132 | -0.438** | -0.179 | -0.292** | -0.512** | -0.317** | -0.229* | -0.219* | -0.232* | -0.297** |  |
|  | **p** | 0.734 | 0.000 | 0.067 | 0.003 | 0.000 | 0.001 | 0.019 | 0.026 | 0.017 | 0.002 |  |
| **ABTS** | **r** | 0.358 | -0.219* | -0.296** | -0.202* | -0.460** | -0.321** | -0.270** | -0.064 | -0.327** | -0.269** |  |
|  | **p** | 0.344 | 0.025 | 0.002 | 0.039 | 0.000 | 0.001 | 0.005 | 0.524 | 0.001 | 0.006 |  |
| **Flavonoids** | **r** | 0.204 | -0.459** | -0.181 | -0.308** | -0.491** | -0.266** | -0.307** | -0.159 | -0.290** | -0.300** |  |
|  | **p** | 0.598 | 0.000 | 0.064 | 0.001 | 0.000 | 0.006 | 0.001 | 0.109 | 0.003 | 0.002 |  |
| **H2O2** | **r** | -0.499 | -0.373** | 0.371** | -0.587** | -0.270** | 0.161 | -0.441** | -0.370** | -0.529** | -0.129 |  |
|  | **p** | 0.172 | 0.000 | 0.000 | 0.000 | 0.005 | 0.102 | 0.000 | 0.000 | 0.000 | 0.193 |  |
| **MDA** | **r** | 0.269 | -0.156 | 0.051 | -0.128 | -0.075 | 0.079 | -0.144 | -0.126 | -0.245* | 0.248* |  |
|  | **p** | 0.483 | 0.113 | 0.608 | 0.192 | 0.444 | 0.425 | 0.142 | 0.204 | 0.012 | 0.011 |  |
| **CAT** | **r** | 0.792* | 0.012 | -0.369** | 0.419** | 0.106 | -0.085 | 0.289** | 0.369** | 0.432** | 0.158 |  |
|  | **p** | 0.011 | 0.902 | 0.000 | 0.000 | 0.283 | 0.387 | 0.003 | 0.000 | 0.000 | 0.108 |  |
| **SOD** | **r** | 0.808** | -0.593** | 0.196* | -0.578** | -0.422** | -0.040 | -0.426** | -0.418** | -0.513** | -0.259** |  |
|  | **p** | 0.008 | 0.000 | 0.045 | 0.000 | 0.000 | 0.687 | 0.000 | 0.000 | 0.000 | 0.008 |  |
| **POD** | **r** | -0.230 | -0.405** | -0.089 | -0.176 | -0.261** | -0.024 | -0.184 | 0.037 | -0.199* | -0.293** |  |
|  | **p** | 0.552 | 0.000 | 0.367 | 0.073 | 0.007 | 0.805 | 0.060 | 0.713 | 0.042 | 0.003 |  |
| *. Correlation is significant at the 0.05 level (2-tailed). | | | | | | | | | | | | |
| **. Correlation is significant at the 0.01 level (2-tailed). | | | | | | | | | | | | |
