# Supplementary material for: Nitrogen, phosphorus, and potassium requirements to improve Sideritis cypria growth, nutrient and water use efficiency in hydroponic cultivation
Source: Heliyon. 2024 Dec 4;11(1):e40755. doi: 10.1016/j.heliyon.2024.e40755 (PMC11699360; doi:10.1016/j.heliyon.2024.e40755)
Supplement: Multimedia component 5 [file mmc5.docx]

**
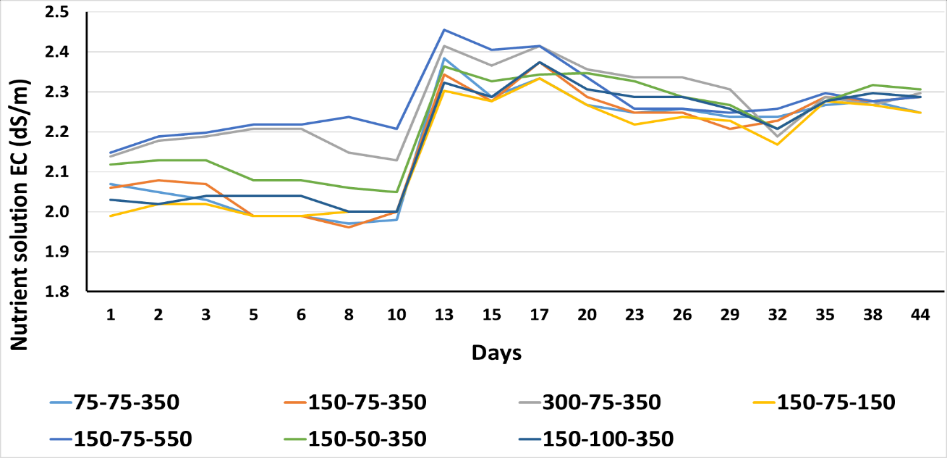

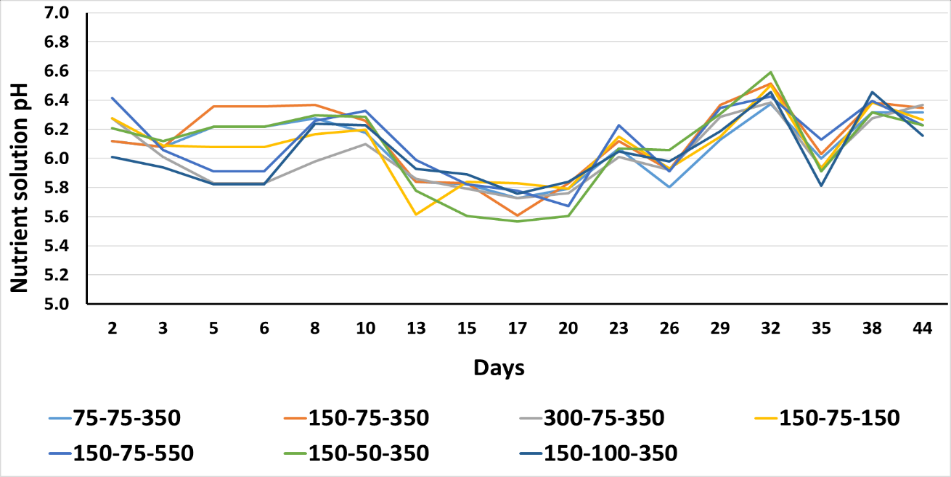
**

**Figure S1**. Effect of nitrogen (N:75, 150, and 300 mg/L), potassium (K: 150, 350, and 550 mg/L), and phosphorus (P: 50, 75, and 100 mg/L) in the nutrient solution in the electrical conductivity (EC: dS/m) and pH of the drainage nutrient solution from *Sideritis cypria* grown hydroponically in NFT system. Error bars show SE.
